# Using cancer survivorship experiences to inform cancer advocacy in Nigeria

**DOI:** 10.3332/ecancer.2025.1887

**Published:** 2025-04-10

**Authors:** Chinelo C Nduka, Chizobam A Nweke, Runcie C W Chidebe, Candidus Nwakasi

**Affiliations:** 1 Department of Community Medicine, Nnamdi Azikiwe University Teaching Hospital, Nnewi, Anambra State, Nigeria; 2 Department of Human Development and Family Sciences, University of Connecticut, Storrs, CT 06269, USA; 3 Department of Sociology and Gerontology, Miami University, Oxford, OH, USA; 4 Scripps Gerontology Center, Oxford, OH, USA; 5 Project PINK BLUE – Health & Psychological Trust Centre, Abuja, Nigeria

**Keywords:** breast neoplasms, health workforce, insurance coverage, medical oncology, cancer treatment outcome

## Abstract

**Background:**

Nigeria has a significant cancer burden, especially breast cancer (BC), which has the highest incidence and mortality in the country. However, like many countries in sub-Saharan Africa, the focus continues to be on infectious diseases to the detriment of addressing non-communicable diseases like cancer with a resultant paucity in available funding, health infrastructure and even health workforce geared towards cancer care. All these invariably lead to very low access to cancer care, with poor patient outcomes being recorded. The purpose of this study is to explore the experiences of BC survivors who are part of a non-profit cancer advocacy group to highlight important areas of cancer survivorship that can be targeted by cancer advocacy programs and interventions in Nigeria.

**Method:**

We used a qualitative descriptive approach and recruited 19 BC survivors through a purposive sampling method. The participants were engaged in four focus group discussions. Analysis of the data resulted in four main themes with direction implications for cancer advocacy among BC survivors in Nigeria. They include ‘Strengthening care interaction quality’; ‘Addressing delayed presentation’; ‘Expanding access to informal support’ and ‘Alleviating the cost burden of cancer care.’ Various recommendations were made, such as better management of the Nigerian Cancer Health Fund, comprehensive cancer coverage by the National Health Insurance Authority and the need for Nigeria’s National Institute for Cancer Research and Treatment to consider retention and training policies for the oncology workforce in Nigeria. This paper highlights the importance of cancer advocacy, arguing that a better understanding of cancer survivorship can strengthen cancer advocacy in Nigeria. In turn, strong cancer advocacy will help to prioritise cancer issues and mitigate a looming cancer crisis in Nigeria.

## Background

The burden of cancer in Nigeria is a major concern, with breast cancer (BC) having the highest incidence and mortality among females [1–3]. However, like many countries in sub-Saharan Africa (SSA), Nigeria focuses more on infectious diseases to the detriment of addressing an increasing burden of non-communicable diseases like cancer. This paper argues that it is important to strengthen cancer advocacy, which will help to prioritise cancer control and mitigate a looming cancer crisis in Nigeria.

In terms of cancer care access in Nigeria, there are 12 comprehensive cancer care centres (CCCC) in Nigeria [4], and they all experience inadequate workforce issues and a lack of functional medical equipment [5]. Access to cancer care is further complicated by social and institutional factors such as poverty, treatment delay, distance to healthcare facilities, unavailability of chemotherapy medications, low budgetary allocation and lack of political will to support cancer control and interventions [6–9]. Further, clinical oncology training in Nigeria is still in its formative years with less than 80 clinical oncologists in the field, [10, 11] and palliative care for cancer is not well established due to weak policy, failure to incorporate palliative care into the medical curriculum and unavailability of medications [12, 13].

For those who access cancer care in Nigeria, there is the issue of delayed presentation. This problem increases the likelihood of poor treatment outcomes and/or mortality [3, 14]. Traditionally, delays in healthcare settings may be due to the patient, healthcare providers or both. Studies have reported high levels of both types of delay in Nigeria [14–16]. Delay may result from poor public knowledge and awareness about cancer and how to recognise the signs and symptoms of cancer, [17] absence of national screening programs, [14] cancer myths, ignorance, cultural beliefs and practices, knowledge deficits and weak referral system [16]. For female BC patients, domestic duties, cost and cancer stigma are factors influencing delayed presentation [15]. More so, some Nigerians consider cancer to be a death sentence, a spiritual attack or a disease beyond modern medicine and may not bother to present early to the hospital – they opt for unverifiable alternative medical solutions, including spiritual rituals. When these practices fail, they would only seek medical care as a last resort [18, 19].

As indicated earlier, the cost of cancer care limits early presentation. For example, the BC financial burden is more than the average SSA family can bear [20]. Seeking BC care in Nigeria, where health insurance is lacking, is largely borne through out-of-pocket payments, [21] and according to Knapp *et al* [22] and Esiaka *et al* [18], many households may have to borrow money to pay for specialised cancer care. In many cancer cases, those who are employed are lower income and cannot afford to pay their medical bills, and those who are self‐employed cannot maintain their source of income because of their illness; consequently, many cancer patients in the country must rely on support from friends and relatives [18]. Therefore, the high cost of cancer treatment may result in delayed presentation or become catastrophic after treatment has commenced, leading to the inability to continue or complete the recommended treatment [23].

### The role of advocacy in cancer care

Historically, cancer advocacy groups have immensely contributed to raising public awareness, promoting education and political activism and raising funds for cancer research [24]. However, these gains have primarily been recorded in developed nations, and Africa needs to catch up. This is because cancer advocacy needs more popularity across the continent. About 10 years ago, Odedina *et al* [25] stated that cancer advocacy is a novel African concept, and Segal *et al* [26] attributed the low priority accorded to cancer in the continent to weak advocacy endeavours. While the literature on cancer advocacy in Nigeria is scanty, there is evidence of government-funded advocacy [27] and advocacy by non-profit groups [28]. Busolo and Woodgate [29] opined that advocacy is needed to build strategic partnerships to ensure cancer prevention and control remain a priority on African governments’ budgets. Odedina *et al* [25] concluded that cancer control planning and implementation cannot be successful without advocacy. The six areas needing direct advocacy include education, politics, research, funding, support and community outreach [26]. This paper highlights the importance of cancer advocacy and argues that a better understanding of cancer survivorship can strengthen cancer advocacy in Nigeria.

Over the past two decades, cancer support groups have helped to address the psychosocial needs of cancer patients and their families, including assisting patients to cope with cancer diagnosis and treatment [30]. Support groups can metamorphose into advocates as different people affected by cancer come together to raise awareness of their cancer challenges. In Nigeria, some non-profit organisations are presently involved in cancer advocacy and organising cancer support groups for people with ongoing cancer survivorship experiences [18]. Orji *et al* [28] found that support groups have made cancer survivors more knowledgeable about their diagnoses and provided them with a platform to make their diagnoses public. Asuzu *et al* [31] also found reduced incidences of distress and improved functional and emotional well-being among BC survivors in Nigeria following interaction with support groups. It is also important to consider support groups for informal cancer caregivers in Nigeria as they share in the cancer burden out of familial commitment or due to cultural expectations [32–34]. The purpose of this study is to explore the experiences of BC survivors who are part of a non-profit cancer advocacy group to highlight important areas of cancer survivorship that can be targeted by cancer advocacy programs and interventions in Nigeria.

## Methods

### Study design

The study used a qualitative descriptive approach to identify participants’ experiences and perceptions relevant to a studied phenomenon with health implications. This approach involves low-inference interpretations of participants’ views about a phenomenon that requires more understanding [35, 36]. The study is also guided by an interpretive epistemological perspective, which assumes individuals have multiple interpretations of a particular event. Therefore, they make sense of their experiences in varied ways [37].

### Research setting and recruitment

The lead researcher travelled to Abuja, Nigeria, to conduct the study. A collaboration between the researchers and a cancer non-profit organisation that runs a cancer psychosocial support centre was instrumental in recruiting participants who met the inclusion criteria. The cancer support group in the psychosocial support centre includes BC survivors, other types of cancer survivors, patient navigators and psychologists. Purposive sampling was used to recruit participants most suitable for addressing the study purpose. The following were the inclusion criteria: adult women (18 years or older), BC survivors, have started or completed any cancer therapy, residing in Nigeria and can communicate in English.

Two women who identified as cancer survivors and members of the cancer support group assisted with the recruitment – they contacted potential participants about the study. They requested their approval to share their contact information with the lead researchers. The PI shared more details about the study and scheduled a date for the focus group discussions (FGD) for those who indicated interest in participating. Verbal informed consent was obtained from all the eligible participants. Ethical approval for the study was given by Providence College Institutional Review Board, Rhode Island, USA and the study was performed according to the principles of the Declaration of Helsinki.

### Data collection

FGD was used to collect data in the study. FGD is useful for collecting quality data about a specific health topic of interest among people who share similar relevant backgrounds. FGDs also yield insightful data because participants can think about their views and experiences within the context of the shared views and experiences of other participants [38]. An FGD guide that was developed with open-ended questions informed by individual interviews conducted was used to explore the cancer survivorship experiences of women cancer survivors in Nigeria. Some of the questions in the FGD guide explored views about access to chemotherapy and fear of chemotherapy, the influence of family members on seeking or continuing treatment (e.g., mastectomy), support from churches and religious leaders in managing cancer and cancer stigma. The richness of the data collected was increased because most of the participants were used to sharing their experiences in support groups, were among familiar people they trust and some had been involved in a previous related study conducted by the research team. Four FGD were conducted in 1 day, and each audio was recorded. The recorded sessions of the FGDs lasted 42 minutes for FGD1, 40 minutes for FGD2, 43 minutes for FGD3 and 32 minutes for FGD4. Informed consent was obtained from all the participants, who were informed of the confidentiality limit in FGD.

### Data analysis

All four audio recordings of the FGDs were professionally transcribed in Nigeria and uploaded to Dedoose (version 9.0.85) for data management. Braun and Clarke’s [39, 40] reflexive thematic analysis approach was used for the data analysis. This approach allows flexibility in identifying patterns within the data, including similar or diverse perspectives of focus group participants, and at the same time, acknowledges the unique role of the researcher in interpreting identified patterns. All the data were analysed using an inductive approach (i.e., data-driven) [41].

The first step involved two of the research team (the first and last author) with diverse professional training (public health, gerontology and medicine) thoroughly reading through the transcripts multiple times to generate a codebook. Second, initial codes were later developed to represent relevant ideas from the data that are best suited to address the research question. Third, the initial codes were reviewed to identify emerging patterns, after which these codes were grouped into sub-themes. Finally, after further review and discussion of the emergent sub-themes based on the research purpose, the researchers reached a consensus to merge relevant sub-themes to form larger themes. To strengthen the trustworthiness of the findings during the analysis and report writing, the researchers sought expert peer review from a well-known cancer patient advocate and regularly had meetings to discuss techniques used and emerging findings to ensure the findings were appropriate for the study.

## Results

The study’s participants (*N* = 19) were BC survivors, ages ranging from 29 to 55. FGD1 had six participants, FGD2 included five, FGD3 had five participants and FGD4 had three. See Table 1 for more participant information.

The data analysis resulted in four main themes with direction implications for cancer advocacy among BC survivors. They include* ‘Strengthening care interaction quality’; ‘Addressing delayed presentation’; ‘Expanding access to informal support’ and ‘Alleviating the cost burden of cancer care.’*

### Strengthening care interaction quality

The data analysis identified that an area requiring advocacy is the need to strengthen care quality in medical settings by improving communication during patient encounters. Many of the participants expressed marked dissatisfaction with the quality of care they received in hospitals. For example, when it came to receiving ‘bad news,’ the way the news of confirming a cancer diagnosis was broken to them resulted in psychological distress. According to Nneka from FGD4, a doctor said to her friend:

I don’t know what to do with you; you have cancer; it has gotten to the spine, it has gotten to the breast; just go back home and be chewing herbs.

For some, cancer care delivery could be improved by paying attention to expressed concerns and ensuring patients and their caregivers had adequate information to help them make informed decisions on treatment modality. For example, Chioma from FGD1 described:

As soon as I had a lump in my breast, my breast was still perfect when they cut off my breast, there was nothing wrong with my breast. It was in the hospital that I found out that people’s breast gets ulcerated, so it seemed as if it was a foolish thing to have done that.

Participants also explained that low cancer care satisfaction discourages hospital care utilisation, which increases the likelihood of adverse outcomes due to cancer. For example, support is needed to address stigmatisation in care settings as they make cancer patients feel less than and less likely to return for care in encounters that affected their self-esteem. One of the participants, Nonye from FGD1, returned to the hospital where she had a mastectomy for a wound dressing, and the nurse refused to care for her. She reported:

I cried because she treated me like a leprosy patient. So, because of that, I refused to go there again for the treatment. It was my husband and I that were now doing the treatment ourselves, and unfortunately, because of that, the thing did not [heal properly] … it affected me; I even ended up in the emergency ward because of that because I refused to go there.

### Addressing delayed presentation

Another theme from the analysis is the need to raise advocacy on social issues that need to be addressed to help mitigate the delayed presentation of cancer. This is because there is the issue of alternative medicine competing with orthodox cancer treatments in the care setting. Sometimes, due to affordability, fear of surgery and chemotherapy, feelings about exposing one’s private body parts or living without one and/or faith in alternative medicine, participants may refuse treatments such as chemotherapy or radiotherapy for alternative cancer therapies that have not been scientifically verified to be effective. Some cancer patients may be fortunate to return to the hospital early enough to still commence treatment, while some may have a markedly advanced stage of cancer that is not amenable to treatment at the time of return. The comment made by Ngozi from FGD1 further explains this theme:

I went to [cancer] hospital, bought a card, and the doctor said it’s still at an early stage, that I should come so that he’ll remove the thing [cancer] since it was still inside the closet. I had not undergone any surgery before; the story I heard about cutting the breast was too alarming and the fear was all over me. So, I ran away for 2 years and started looking for a native cure. Eventually, I returned to the hospital.

Just like Ngozi from FGD1, Oge from FGD3 shared, ‘The issue a lot of young women have is ‘if I cut my breast, who will marry me’? If I cut my breast now that I’m young, my husband will go [not be able to find husband].’ The above comments also suggest that some women with BC were reluctant to present to the hospital because surgically removing the breasts is an overwhelming encounter that they do not want to experience despite their illness. Thus, many of them, especially younger women, are concerned with the consequences (e.g., attractiveness, ability to find a husband and breastfeeding a baby) of some treatment modalities for BC, especially mastectomy.

### Expanding access to informal support

This theme described the role of informal support from family and friends as an essential backbone of cancer care in a resource-challenged setting such as Nigeria. Family support can be in the form of providing a buffer against the risk of psychological distress from cancer, thus helping with cancer coping. Social support from family and friends also alleviates the difficulty of going through treatment. According to Onyinye from FGD1:

I think that’s [support] one great thing that the patient needs. When you don’t have that support from your family or friends, that might even kill you before the disease itself. I used to wonder how those that don’t have anyone around cope with this kind of problem.

In addition to family and friends’ informal support in the form of psychosocial support, such as encouraging the cancer survivors, accompanying them to the hospital and making efforts to reduce their risk of loneliness and hopelessness, participants also highlighted the empowerment to decide and take action that comes from having supportive friends and relatives. This is especially important when there is lack of a collective family support, and this can limit care-seeking behaviour. For example, family members who cancer survivors depend on for financial support may think that late-stage cancer does not deserve to pursue treatments because it is a waste of resources. Amaka from FGD2 said:

In my situation, a friend of my husband advised him not to spend money and that I’d die after everything. So, my husband was holding back his money, looking at me and waiting for me to die. It was my brother who took it up and helped me.

Similarly, Ndidi from FGD1 added:

So, we were not seeing a doctor; my husband had this mindset because his people had told him not to allow me to do chemo [chemotherapy]. If not because I have very strong support from my own side, I would have fallen victim; I would have been left at the mercy of ‘whatever happens’, but my people said no, that I would do the chemo and we’ll pray and that was it.

### Alleviating the cost burden of cancer care

This theme, alleviating the financial burden of cancer care, is another advocacy focus identified from our data analysis. Participants reported that the cost of cancer treatment has made many people unable to commence treatment, and even some cancer survivors have had to discontinue treatment due to the high, unaffordable cost. The cost of cancer care is worsened by the fact that most of the participants paid out-of-pocket for medical treatment. For most, the ability to pay out-of-pocket includes seeking financial support from others and religious organisations like churches and mosques. Unfortunately, this may be unsuccessful for some as potential sources of financial support see the money requested as too steep for a treatment that they do not think will be effective in curing cancer. Ify from FGD1 explained:

I went to a catholic church because I was going around to look for money, so when I took the prescription to a reverend father, he asked me what is the guarantee that if I took this drug, I would live.

Additionally, because all our study participants were paying out-of-pocket for cancer therapy, the unavailability of funds was reported as a barrier to starting lifesaving treatment. For example, Nkechi from FGD1, who had undergone a round of chemotherapy and radiotherapy before she had a recurrence, explained:

Then, there was a drug that they asked me to buy. I’m just hoping that by God’s grace, I’ll get it because I can’t afford the drug. I’m supposed to take it for 21 days. So, I approached the pharmacy, and they said the drug is six million naira [$6,000], but if I can pay 50%, they will order it…So, since October, I have not been able to take it because I cannot afford it.

Alleviating the cost of cancer care will be important for improving patients’ outcomes, especially if they can complete the course of their chemotherapy. One participant, Ify from FGD1, had recognised the positive effect of a targeted therapy she was placed on and was disheartened that she could not afford the complete dosage of the treatment course. She said:

I collected the last bone scan result, and it showed me that the particular [tumours] I was having in some places were no longer there, but in some other places, it is still there. So that means that if I continue with the targeted therapy, things will normalise, but there’s no money. I’m a civil servant and a junior one, for that matter, and I have two children in medical school. How do I cope with that?

## Discussion

The study explored the experiences of BC survivors to highlight important areas of cancer survivorship that can be targeted by cancer advocacy programs and interventions in Nigeria.

Our study findings show that the quality of care received by this group of Nigerian BC survivors was poor, which is unsurprising given that the basic infrastructure needed for cancer care and treatment in Nigeria is lacking. Cancer control in Nigeria deserves high priority as the burden of cancer in Nigeria is increasing rapidly, and advocacy plays a major role in raising awareness and channelling public and private resources and policies toward cancer control. To reduce cancer burden, improve patient treatment outcomes and strengthen oncology services in this population, our findings showed interesting areas of advocacy for the government, non-profits and other stakeholders as explored by the participants.

Alleviating the cost burden of cancer care in Nigeria was a central advocacy need for cancer survivors. The financial cost of BC treatment in Nigeria is a major cause for concern as it is currently overwhelming given the predominant out-of-pocket mode of payment for cancer care. Like this study, other studies [21, 23] have also shown that the cost of treatment has led to delays in the commencement of treatment and even abandoned treatments. There is a need for improved funding of cancer in Nigeria, and it has been noted that the most cost-effective way of reducing cancer burden is by enhancing awareness and the implementation of prevention programs [20].

The catastrophic health expenditure associated with cancer treatment in Nigeria has been a persistent advocacy issue without much success. In 2019, the Nigerian Federal Ministry of Health (FMoH) launched the Cancer Health Fund (CHF) and budgeted N729 million ($7.2 million) to provide funding to indigent cancer patients and access to standard cancer treatment. The CHF is still being piloted to provide some care and treatment for people with breast, prostate and cervical cancers in six CCCC, one in each geopolitical zone of Nigeria [42]. While this is laudable progress, the CHF is still marred with poor implementation, including limited access to the fund, non-representation of the patients, unclear eligibility criteria and weak accountability. We argue that the National Health Insurance Authority (NHIA), which is legally mandated to regulate, supervise and manage health insurance [43, 44], is better positioned to manage the CHF than the FMoH, which currently manages and disburses the funds through the six CCCC. If the CHF is moved to the NHIA, it would be an important step towards increasing insurance coverage and timely access to cancer care.

In addition, we propose the need for sustained policy advocacy for the NHIA to cover comprehensive cancer care for several reasons. First, the full coverage of comprehensive cancer care by the NHIA will increase the coverage of cancer patients, and such inclusion in the NHIA at the Federal level would serve as a guideline that State Governments could follow for their state health insurance schemes and even private health insurance schemes with significant coverage of Nigerian cancer patients. Second, the chemotherapy market in Nigeria features a fragmented supplier landscape, low volumes, variable quality and a lack of transparency in pricing [3]. If cancer treatment becomes a fully covered service under NHIA, the pooling of resources will lead to the identification of treatment regimens that produce similar outcomes but at significantly lower costs, engagement of top-quality generic drug manufacturers and large volume procurement of these drugs, thus guaranteeing the availability of quality drugs at cheaper rates. Finally, we know that cancer care and treatment are expensive, and there is a controversy that full coverage of cancer care by the NHIA will deplete the funds of the authority; we argue that the new NHIA Bill 2022, which was signed into law by President Muhammadu Buhari proposed the Vulnerable Group Fund which targets older people, the poor and other vulnerable people [44]. Cancer patients and survivors will remain the most vulnerable groups if they continue to be excluded from full NHIA coverage.

Nigeria’s oncology care is faced with multiple challenges which have been compounded by the shortage of healthcare workforce, [17] poor oncology specialty training and high clinical oncology workload [10]. While healthcare workforce shortage is a global challenge, high-income countries are addressing their shortages by recruiting health workforce from low and middle-income countries like Nigeria, further worsening the situation [17]. Our study findings showed that strengthening the care interaction quality is another important advocacy area of interest for BC survivors as they would like to have their oncology team spend quality time communicating their condition and treatment plans. Arguably, addressing the clinical oncology specialists’ shortage may reduce workload, thereby increasing the time the oncology team spends communicating with patients. Therefore, Nigeria’s National Institute for Cancer Research and Treatment needs to urgently consider retention and training policies for the oncology workforce in Nigeria. Again, the oncology health workforce is relatively complicated as cancer patients are attended to by numerous healthcare providers, including surgeons, medical oncologists, pathologists, radiation oncologists and many others across the continuum of care. Thus, we also propose innovative treatment guidelines for simple, clear and understandable cancer patient-healthcare provider communication and patient navigation.

Of utmost importance is to address the widespread ignorance about cancer among the population as cancer awareness is especially important to improve risk reduction behaviours, promote timely cancer screening for early detection and ultimately reduce the burden of cancer [17]. Such awareness helps to dispel the prevailing myths and misconceptions about cancer in the population, which is a significant factor in the delay in presentation by cancer survivors with associated worsened outcomes [18, 23]. There is a vicious cycle of delay in presentation for treatment due to widespread misconception that cancer is a death sentence that they will not survive, then the pains and worsening condition ultimately make them present when the cancer is already at an advanced stage and cure is not feasible, [33] such that they do not survive, which only serves to buttress the misconception and so the cycle continues. Communication channels like television, radio, press, social and print media can be used to pass correct information about cancer to the population. In addition, religious and traditional leaders, including other influential stakeholders, can be engaged to assist in the education of their communities or members through campaigns that provide evidence-based information about cancer, its risk factors, prevention and early detection. This can also help raise awareness and increase the uptake of available cancer screening programs, which would promote earlier detection and better survival rates. Another low-hanging fruit is the organisation of a well-coordinated national screening program, [45] which is non-existent currently, that promotes early diagnosis and identification of cancer at an early stage when treatment is more effective and less expensive.

Organisation of cancer support groups for cancer survivors is also a very important aspect of cancer care as it has been documented to help cancer patients cope with cancer treatment and care, thus improving their chances of survival. This is because, in these groups, they identify people with similar stories and experiences who will listen to them, understand and inspire them [18]. For example, a BC patient who meets a cancer survivor who is living well and optimistic about their life may be inspired to look forward to a full recovery. Although very few studies have considered gender differences in cancer survivorship, the findings from this study revealed that many BC patients had to rely on their husbands to approve their treatment before commencement, which negatively affected some women’s outcomes if the man was not responsive. This could be a pointer to economic abuse whereby the husband controls all the resources in the house, and the woman is unable to access them without his express permission [46].

Cancer advocacy groups such as Project PINK BLUE – Health and Psychological Trust Centre, Sebeccly Cancer Care, CancerAware Nigeria, Exquisite Magazine Cancer Care Foundation, Efferent Cares and many others have been galvanising actions against cancer through public education, and patients’ support with reported success according to the Landscape Study of NGO-Led Cancer Interventions in Nigeria. These are important advocacy needs, especially public education, whether formal or informal, on cancer and cancer control to create awareness about the burden of cancer and its risk factors, address some persisting misconceptions about cancer which are usually responsible for the stigma faced by cancer patients, educate the public on the importance of screening for early detection and treatment including how different treatment modalities can be complementary rather than exclusive.

## Limitation, strengths and future directions

The use of a qualitative descriptive approach was a strength in this study as it provided insightful and rich meaning into the experiences of the survivors and where they hope to see cancer advocacy in Nigeria. However, the results of this study should be interpreted with caution as we used a reflective thematic analysis, which provided researchers with the opportunity to offer interpretation. Also, the inclusion of only patients who can speak English is a limitation. With this inclusion criteria, we excluded the voices of local Nigerian BC survivors whose voices are rarely considered in research. Future research should consider an explorative study with survivors of other cancers because advocacy needs of BC survivors may differ greatly from the need of other cancers.

## Conclusion

This paper highlights the importance of cancer advocacy, arguing that a better understanding of cancer survivorship can strengthen advocacy in Nigeria. In turn, strong advocacy will help prioritise cancer control and mitigate a looming cancer crisis in Nigeria.

## Conflicts of interest

None to declare.

## Funding

No funding was received for this study.

## Author contributions

CCN: Conceptualisation, design, writing of manuscript and review of the manuscript; CAN: Writing of manuscript and review of the manuscript; RCWC: Manuscript draft revision and review of the manuscript; CN: Conceptualisation, data collection, design, writing of manuscript and review of the manuscript. All authors read, reviewed and approved the final revision of the manuscript.

## Figures and Tables

**Table 1.** Demographic characteristics of participating BC survivors.

Participants	Age	Marital status	Education	Therapy status	Year of diagnosis
19 Women	29–55	7 Married 5 Single 3 Separated/Divorced 1 widow 2 Unknown	15 Tertiary 1 High School 2 Unknown	11 Completed. 6 Ongoing 2 Unknown	5 in 2017 3 in 2019 2 in 2018 2 in 2010 1 in 2014 1 in 2015 1 in 2016 3 Unknown
